# Enhancing Quality through Equity and Access in an Age-friendly Ecosystem

**DOI:** 10.1093/geroni/igaf122.4398

**Published:** 2025-12-31

**Authors:** Robyn Golden, Elizabeth Davis, Walter Rosenberg, Diane Mariani, Alison Wagner, Padraic Stanley, Erin Emery-Tiburcio, Molly Jenkins

**Affiliations:** Rush University Medical Center, Chicago, Illinois, United States; Rush University Medical Center, Chicago, Illinois, United States; Rush University Medical Center, Chicago, Illinois, United States; Rush University Medical Center, Chicago, Illinois, United States; Rush University Medical Center, Chicago, Illinois, United States; Rush University Medical Center, Chicago, Illinois, United States; Rush University Medical Center, Chicago, Illinois, United States; Rush University Medical Center, Chicago, Illinois, United States

## Abstract

Ensuring timely, quality, and culturally responsive access to medical and social care is essential to equity in meeting the diverse needs of older adults. Access influences every aspect of care delivery, from early intervention to ongoing management of chronic conditions, and plays a key role in improving health and wellbeing. This symposium will highlight how programs at Rush University Medical Center are breaking down barriers to care through the 4M’s of an Age-Friendly Health System Framework and expanding opportunities for older adults, caregivers, and providers. Presentations will feature four initiatives that are built on the foundation of the 4Ms of an Age-Friendly Health System: Schaalman Senior Voices expands access to geriatric education for healthcare providers and students to enhance their ability to care for older adults; Falls Prevention initiatives expand community access to education on risks and strategies by addressing social determinants of health and offering English- and Spanish-language sessions; Rush@Home delivers team-based primary and social care directly to patients’ homes; and Caring for Caregivers (C4C) connects caregivers with supportive services that strengthen their capacity to provide care. Speakers will share emerging data and recent program developments, followed by a discussion of practical strategies to expand access to education, caregiver support, coordinated home-based services, and clinical care. The session will conclude with an interactive dialogue offering actionable strategies for clinicians, educators, policymakers, and advocates to take a multi-pronged, holistic approach to ensuring older adults receive quality healthcare.

